# From *in silico* to *in vitro*: discovery and validation of peptide modulators of IL-6/IL-6R signaling in rheumatoid arthritis

**DOI:** 10.3389/fimmu.2026.1794095

**Published:** 2026-04-29

**Authors:** Junping Yang, Yan Wang, Jie Huang, Jing Zhang, Ying Xie

**Affiliations:** 1 Department of General Practice, Wuhu Second People’s Hospital, Wuhu, Anhui, China; 2 Department of Neurology, Affiliated Hospital and Clinical Medical College of Chengdu University, Chengdu, China; 3 University of Electronic Science and Technology of China, Sichuan Provincial People’s Hospital, Department of Rheumatology and Immunology, School of Medicine, Chengdu, China; 4 Department of Orthopedics, Affiliated Chuzhou Hospital of Anhui Medical University (The First People’s Hospital of Chuzhou), Chuzhou, Anhui, China

**Keywords:** artificial intelligence, immunoinformatics, interleukin-6 receptor, molecular docking, molecular dynamics, peptide therapeutics, rheumatoid arthritis, STAT3 signaling

## Abstract

**Background:**

Rheumatoid arthritis (RA) is a chronic autoimmune disease in which dysregulated interleukin-6 (IL-6) signaling through the IL-6 receptor (IL-6R) plays a central pathogenic role. Although monoclonal antibodies targeting this pathway are clinically effective, their use is limited by parenteral administration, high cost, and systemic immunosuppression. Peptide-based inhibitors represent a complementary strategy for modulating cytokine–receptor interactions, offering advantages in design flexibility and manufacturability. Recent advances in immunoinformatics and artificial intelligence (AI) facilitate the rational identification of peptide candidates with favorable safety profiles.

**Methods:**

We developed an integrated AI-assisted discovery pipeline incorporating immunoinformatics-based safety screening (toxicity, allergenicity, and antigenicity prediction), structural modeling, molecular docking with MM/GBSA rescoring, and 100-ns molecular dynamics (MD) simulations to identify IL-6R-targeting peptides. Seven candidate peptides (P01–P07) were prioritized based on predicted safety, binding energetics, and structural stability. Lead candidates were experimentally evaluated using competitive ELISA assays for IL-6/IL-6R binding and cell-based assays measuring IL-6–induced STAT3 phosphorylation.

**Results:**

Computational analyses consistently identified peptide P01 as the top-ranked candidate, exhibiting stable binding conformations, persistent hydrogen bonding at the IL-6R interface, low RMSD and RMSF values during MD simulations, and the most favorable MM/GBSA binding free energy. *In vitro* experiments confirmed that P01 competitively inhibited IL-6/IL-6R binding (IC_50_ ≈ 1.6 µM) and suppressed IL-6–induced STAT3 phosphorylation without detectable cytotoxicity. The strong concordance between computational predictions and experimental findings supports the robustness of the discovery workflow.

**Conclusion:**

This study establishes a safety-focused, AI-driven peptide discovery framework integrating in silico prioritization with experimental validation. Peptide P01 represents a tractable early-stage IL-6R antagonist that provides a foundation for future structure-guided optimization and development as a complementary therapeutic modality for IL-6–driven inflammatory diseases.

## 1 Introduction

Rheumatoid arthritis (RA) is a systemic autoimmune disease characterized by chronic synovial inflammation, progressive joint destruction, and substantial extra-articular morbidity (1). Affecting approximately 0.5–1% of the global population, RA remains a major cause of long-term disability and impaired quality of life despite continued advances in therapeutic management (2). Estimates from the Global Burden of Disease (GBD) Study 2021 indicate that RA accounts for more than three million disability-adjusted life years worldwide, highlighting its persistent prevalence and considerable socioeconomic burden (3). Although conventional disease-modifying antirheumatic drugs (DMARDs), such as methotrexate and sulfasalazine, remain first-line therapies, and biologic and targeted synthetic DMARDs have broadened treatment options, a substantial proportion of patients experience inadequate response, intolerance, or loss of efficacy over time (4). This ongoing treatment gap underscores the need for additional and complementary therapeutic strategies.

Among the cytokines implicated in RA pathogenesis, interleukin-6 (IL-6) and its receptor (IL-6Rα) play a central role in mediating both local joint inflammation and systemic disease manifestations (5). IL-6 promotes synovial immune activation, B-cell differentiation, Th17 polarization, and osteoclastogenesis, while also contributing to systemic features such as anemia of chronic disease, fatigue, and fever (6). IL-6 signaling is mediated through two principal pathways: classical signaling via membrane-bound IL-6Rα and trans-signaling involving soluble IL-6Rα in complex with the ubiquitously expressed gp130 receptor (7). Dysregulation of these signaling routes sustains inflammatory cascades within synovial tissue and reinforces disease chronicity, establishing IL-6/IL-6R as a validated therapeutic target in RA (8).

Although IL-6 participates in a variety of inflammatory and oncogenic processes, its role in rheumatoid arthritis is particularly prominent. Elevated IL-6 levels are consistently detected in synovial fluid and serum of RA patients and correlate with disease activity, joint destruction, and systemic manifestations such as fatigue and anemia. IL-6 signaling promotes differentiation of Th17 cells, activation of B cells, and osteoclastogenesis, all of which contribute to persistent synovial inflammation and bone erosion in RA. These disease-specific mechanisms have established IL-6 and its receptor as validated therapeutic targets, as demonstrated by the clinical success of IL-6R-targeting monoclonal antibodies.

Several biologic agents targeting inflammatory cytokines have transformed RA treatment, including TNF inhibitors such as etanercept and IL-1 receptor antagonists such as anakinra. However, these therapies target specific inflammatory mediators that may not fully capture the broader regulatory role of IL-6 in immune signaling. IL-6 signaling integrates multiple inflammatory pathways, including activation of the JAK/STAT3 cascade, regulation of acute-phase responses, and modulation of adaptive immune responses. Consequently, IL-6 blockade has demonstrated clinical benefit even in patients who exhibit inadequate responses to TNF inhibition. The peptide-based strategy proposed in this study aims to modulate the IL-6 receptor interface through competitive binding, offering a complementary molecular approach to cytokine inhibition. While monoclonal antibodies remain highly effective therapeutics, peptide inhibitors may provide advantages in terms of molecular tunability, manufacturing scalability, and potential reduction of immunogenic responses, although these aspects require further investigation.

Monoclonal antibodies targeting IL-6 or IL-6R, including tocilizumab and sarilumab, have demonstrated robust clinical efficacy, leading to improvements in disease activity, functional outcomes, and radiographic progression in patients with RA (4, 9). However, their clinical use is constrained by several limitations, including parenteral administration, high manufacturing and storage costs, increased susceptibility to infection associated with systemic immunosuppression, immunogenicity, and primary or secondary non-response in a subset of patients (10, 11). Although the introduction of biosimilars has partially alleviated cost-related barriers, access to IL-6-targeted biologic therapies remains uneven across healthcare settings (12). These considerations continue to motivate the exploration of alternative and complementary therapeutic modalities that may enhance flexibility and scalability while preserving efficacy.

Peptide-based therapeutics have emerged as promising scaffolds for modulating protein–protein interactions, including cytokine–receptor interfaces (13). Unlike small molecules, which often struggle to engage large or shallow binding surfaces, peptides can mimic native interaction motifs, enabling high specificity and favorable binding complementarity (14). Compared with antibodies, peptides are smaller in size, which may facilitate improved tissue penetration and support alternative formulation strategies (15). Advances in peptide chemistry—including cyclization, incorporation of non-natural amino acids, lipidation, and PEGylation—have substantially improved peptide stability, bioavailability, and pharmacokinetic properties (16, 17). Notably, the development of PN-2921, a PEGylated IL-6-binding peptide with high potency in preclinical models, provides proof-of-concept that peptides can effectively modulate IL-6 signaling (18).

The feasibility of peptide therapeutics has further been reinforced by advances in immune checkpoint modulation and cytokine inhibition. Peptide inhibitors targeting PD-1/PD-L1 interactions have demonstrated immune-modulatory activity in preclinical cancer models (19), while peptide-based approaches directed against IL-1β and TNF signaling are under active investigation for inflammatory diseases (20, 21). Collectively, these studies support the broader applicability of peptide scaffolds as modulators of immune signaling pathways.

In parallel, computational and AI-driven methodologies have transformed peptide discovery. Deep learning-based structure prediction platforms and machine-learning models trained on peptide–protein interaction data enable systematic identification and prioritization of candidate sequences (22, 23). Integration of these approaches with molecular docking, MM/GBSA free-energy estimation, and molecular dynamics simulations allows high-resolution assessment of binding affinity, conformational stability, and interaction persistence (24). In addition, immunoinformatics-based safety filters—such as predictions of toxicity, allergenicity, and antigenicity can reduce downstream attrition by excluding unfavorable candidates early in the discovery process (25, 26).

Building on these advances, the present study implements an integrated, safety-focused, AI-assisted pipeline combining immunoinformatics screening, structural modeling, molecular docking, molecular dynamics simulations, and experimental validation to identify peptide inhibitors of IL-6R. Using this framework, we prioritized a limited set of candidate peptides and identified Peptide-1 (P01) as a competitive inhibitor of IL-6/IL-6R interaction *in vitro*. These findings establish a rational foundation for peptide lead identification and provide a basis for subsequent structure-guided optimization and preclinical evaluation in IL-6-driven inflammatory diseases.

## 2 Materials and methods

**Figure 1:**
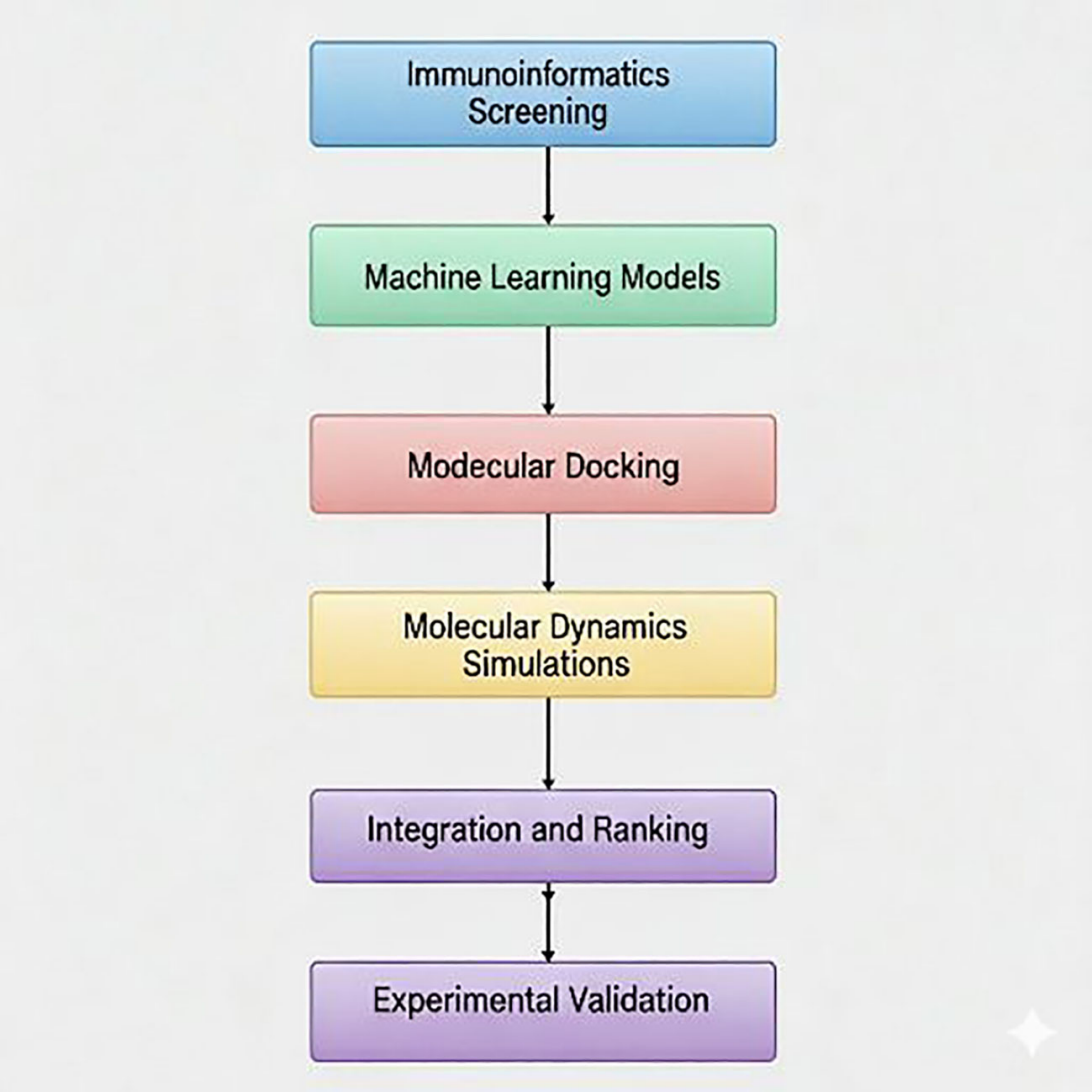
AI-assisted workflow for discovery and in vitro validation of IL-6R-targeting peptides. Integrated pipeline illustrating immunoinformatics filtering, machine-learning–based IL-6 induction classification, structure-based docking, molecular dynamics simulations, multi-criteria ranking, and experimental validation of peptide candidates.

All experimental procedures were conducted following established protocols for molecular and cellular assays to ensure reproducibility and reliability. Protein-level analyses were performed using complementary immunoassays, including enzyme-linked immunosorbent assay (ELISA) and Western blotting, which enable sensitive and specific detection of cytokine–receptor interactions and intracellular signaling events through antigen–antibody binding and enzymatic readouts **Figure 1**. All experiments were performed with at least three independent biological replicates, with technical replicates included where appropriate.

### 2.1 Machine learning–based immunomodulatory peptide filtering

A curated dataset of experimentally annotated peptides was obtained from the Immune Epitope Database (IEDB; accessed March 2024), comprising 3,356 non-redundant sequences, including 365 IL-6–inducing and 2,991 non-inducing peptides. The machine-learning framework was designed as a safety-oriented filtering step to prioritize peptides with low IL-6–inducing potential rather than directly predicting receptor binding affinity.

Peptide sequences were encoded using a hybrid feature representation combining one-hot encoding and physicochemical descriptors. Each amino acid residue was mapped to a 20-dimensional binary vector, while additional features captured hydrophobicity, steric effects, molecular volume, and polarizability. The dataset was preprocessed by binarizing binding indices using a threshold of 0.5, followed by partitioning into training (80%) and testing (20%) subsets. Continuous features were normalized using Z-score scaling to improve model stability.

Five classification algorithms—Logistic Regression, K-Nearest Neighbors (KNN), Random Forest, Gradient Boosting, and XGBoost—were evaluated. XGBoost was selected as the primary model and optimized using binary cross-entropy loss, with class imbalance addressed through the scale_pos_weight parameter. Model performance was assessed using accuracy, precision, recall, F1-score, ROC-AUC, and PR-AUC, with five-fold cross-validation applied to evaluate generalization. Model calibration was further examined using the Hosmer–Lemeshow test, and feature importance was interpreted using SHAP (SHapley Additive exPlanations).

Predicted residue-level probabilities were aggregated to generate peptide-level scores, including mean probability, maximum probability, and top-3 mean probability, enabling prioritization of candidate peptides with favorable safety profiles.

For a peptide sequence P={a1,…,an}and the feature representation was:


$$F(P)=\{f(a_{1}),f(a_{2}),\ldots ,f(a_{n})\},\;F\in {\mathbb{R}}^{n\times d}$$

where f(ai) is the feature vector of residue ai.

Binding indices were binarized into two classes using a threshold of 0.5:


$$y_{i}=\{\begin{array}{ll} 1, & \text{if }BI_{i}\geq 0.5\\ 0, & \text{if }BI_{i}<0.5 \end{array}$$

Datasets were divided into features (X) and targets (y) and partitioned into training (80%) and testing (20%) subsets. Z-score normalization was applied to continuous feature descriptors to improve numerical stability and model convergence during training.

Five classifiers were evaluated: Logistic Regression, K-Nearest Neighbors (KNN), Random Forest, Gradient Boosting, and XGBoost. XGBoost was optimized as the primary model using binary cross-entropy loss:


$$L=-\frac{1}{N}\sum_{i=1}^{N}\left[y_{i}\;\log({\hat{y}}_{i}\right)+(1-y_{i})\log(1-{\hat{y}}_{i})]$$

Class imbalance was addressed via the scale_pos_weight parameter:


$$\text{scale}\_\text{pos}\_\text{weight}=\frac{N_{\text{negative}}}{N_{\text{positive}}}$$

Performance metrics included accuracy, precision, recall, F1-score, ROC-AUC, and PR-AUC. The F1-score was defined as:


$$F1=\frac{2\cdot \text{Precision}\cdot \text{Recall}}{\text{Precision}\cdot \text{Recall}}$$

Five-fold cross-validation was used for generalization. Model calibration was assessed using complementary goodness-of-fit metrics, including the Hosmer–Lemeshow statistic. SHAP (SHapley Additive exPlanations; https://shap.readthedocs.io/) was applied to interpret feature contributions.

Predicted residue-level class probabilities derived from the trained classifier were aggregated at the peptide level:


$$\bar{p}=\frac{1}{n}\sum_{i=1}^{n}p_{i}$$

where pi is the residue probability. Maximum and top-3 mean probabilities were also calculated to emphasize high-affinity residues.

### 2.2 Peptide design and immunoinformatics screening

The human IL-6 receptor (IL-6R) sequence (UniProt ID: P40189) was retrieved from UniProt and used for structural modeling. Candidate peptides were initially generated using the IL6Pred server, which classifies sequences as IL-6 inducers or non-inducers. Peptides with prediction scores below 0.5 were retained as non-inducers.

Shortlisted peptides were further evaluated using immunoinformatics-based safety screening tools. Toxicity was assessed using ToxiPred2, antigenicity using VaxiJen v2.0, and allergenicity using AllerTOP v2.0. Only peptides predicted to be non-toxic, non-allergenic, and low in antigenicity were retained for further analysis. This two-stage filtering strategy ensured the selection of candidates with minimized risk of inducing pro-inflammatory responses.

### 2.3 Structural modeling and docking

Three-dimensional structures of candidate peptides were generated using AlphaFold3 and validated using the SAVES v6.1 server through Ramachandran plot and ERRAT analyses. Only models meeting accepted stereochemical quality criteria were selected for docking studies.

Molecular docking was performed using ClusPro, which applies rigid-body docking and clustering algorithms to identify energetically favorable binding orientations. This approach was used as an efficient initial screening method for peptide–protein interactions across large binding interfaces. Although rigid docking does not fully account for conformational flexibility, subsequent molecular dynamics simulations were employed to refine interaction stability.

The docking interface was defined based on previously reported structural data describing IL-6 binding to the extracellular domain of IL-6R. Docking poses were analyzed to determine whether peptides localized within or near the IL-6 binding interface, suggesting potential competitive inhibition. Interaction profiles, including hydrogen bonds and hydrophobic contacts, were analyzed using LigPlot+.

### 2.4 Molecular dynamics simulations

Molecular dynamics (MD) simulations were performed using GROMACS (version 2022) with the CHARMM36m force field to evaluate the stability and dynamics of peptide–IL-6R complexes. Each system was solvated in a cubic box using the TIP3P water model, with a minimum distance of 1.0 nm between the protein and box edges. Sodium and chloride ions were added to neutralize the system and achieve a physiological ionic strength of 0.15 M.

Energy minimization was conducted using the steepest descent algorithm, followed by equilibration under NVT (100 ps, 300 K) and NPT (100 ps, 1 bar) conditions using V-rescale and Parrinello–Rahman coupling schemes, respectively. Long-range electrostatic interactions were treated using the Particle Mesh Ewald method with a cutoff of 1.2 nm, and bond constraints were applied using the LINCS algorithm with a 2 fs timestep.

Production simulations were performed for 100 ns under periodic boundary conditions. Additional trajectory analyses, including principal component analysis, free energy landscape (FEL), and solvent accessible surface area (SASA), were performed to qualitatively assess conformational stability and structural compactness.

### 2.5 Peptide synthesis and characterization

The top-ranked peptide (P01: TWLVQALFIFLTTES) was synthesized using Fmoc-based solid-phase peptide synthesis on Rink-amide resin. Coupling reactions were performed using HATU/DIPEA chemistry. Crude peptides were cleaved using a TFA: TIS:H_2_O (95:2.5:2.5) mixture, purified by reverse-phase high-performance liquid chromatography (HPLC), and characterized using electrospray ionization mass spectrometry (ESI-MS), high-resolution mass spectrometry (HR-MS), and LC-MS/MS fragmentation.

### 2.6 *In vitro* functional assays

#### 2.6.1 Competitive ELISA for IL-6/IL-6R binding

Competitive ELISA was performed to evaluate inhibition of IL-6 binding to IL-6R. Recombinant human IL-6R (2 µg/mL) was coated onto 96-well plates overnight at 4 °C. Plates were washed and blocked with 5% BSA in PBS to minimize nonspecific binding.

Recombinant IL-6 (10 ng/mL; ~0.48 nM) was pre-incubated with peptide P01 (0.01–100 µM) and added to coated wells. Bound IL-6 was detected using a biotinylated anti-IL-6 antibody followed by HRP-conjugated streptavidin. Signal development was performed using TMB substrate, and absorbance was measured at 450 nm.

Percent inhibition was calculated relative to IL-6-only controls, and IC_50_ values were determined using nonlinear regression. Protein concentrations were reported in both mass and molar units to ensure clarity in ligand–receptor stoichiometry, while peptide concentrations were expressed in micromolar units. The use of excess peptide concentrations was necessary to achieve effective competitive inhibition due to differences in molecular size and binding affinity.

Percent inhibition was calculated as:


$$\%\text{Inhibition}=\frac{OD_{control}-OD_{sample}}{OD_{control}-OD_{blank}}\times 100$$

IC_50_ values were derived by nonlinear regression (GraphPad Prism).

Recombinant human IL-6 (10 ng/mL; ~0.48 nM) was used as a fixed ligand concentration, while peptide concentrations were varied in the micromolar range to enable competitive binding and determination of IC_50_ values.

#### 2.6.2 STAT3 phosphorylation assay

HepG2 cells were used to evaluate IL-6–induced STAT3 signaling. Cells were serum-starved and treated with peptide P01 prior to IL-6 stimulation (10 ng/mL). STAT3 phosphorylation (Tyr705) was assessed using Western blotting and ELISA-based assays, while downstream gene expression (SOCS3 and CCL2) was evaluated using quantitative PCR.

Although HepG2 cells are not disease-specific, they provide a robust and reproducible model for IL-6 signaling. Future studies will extend validation to rheumatoid arthritis–relevant cellular systems.

### 2.7 Western blot analysis of STAT3 phosphorylation

Protein samples were prepared using RIPA lysis buffer (50 mM Tris-HCl, pH 7.4; 150 mM NaCl; 1% NP-40; 0.5% sodium deoxycholate; 0.1% SDS) supplemented with protease and phosphatase inhibitor cocktails. Protein concentrations were quantified using the BCA assay, and equal amounts of protein (20–30 µg per lane) were mixed with 4× Laemmli sample buffer and denatured at 95 °C for 5 min prior to electrophoresis.

Proteins were separated by SDS-PAGE using 10% polyacrylamide gels under reducing conditions. Electrophoresis was performed at 80 V for stacking and 120 V for resolving until adequate band separation was achieved. Following separation, proteins were transferred onto polyvinylidene fluoride (PVDF) membranes (0.45 µm pore size) using a wet transfer system at 100 V for 60–90 min at 4 °C.

Membranes were blocked with 5% non-fat dry milk or 5% BSA in Tris-buffered saline containing 0.1% Tween-20 (TBST) for 1 h at room temperature. Primary antibodies against phospho-STAT3 (Tyr705), total STAT3, and β-actin were incubated overnight at 4 °C at manufacturer-recommended dilutions (typically 1:1000). After washing with TBST, membranes were incubated with HRP-conjugated secondary antibodies (1:5000 dilution) for 1 h at room temperature.

Protein bands were visualized using enhanced chemiluminescence (ECL) reagents and imaged using a gel documentation system. Densitometric analysis was performed using ImageJ software, and phospho-STAT3 levels were normalized to total STAT3, with β-actin serving as a loading control.

### 2.8 Quantitative real-time PCR

Total RNA was extracted using TRIzol reagent, and cDNA was synthesized using reverse transcription kits. Quantitative PCR was performed using SYBR Green chemistry, and gene expression levels were calculated using the 2^-^ΔΔCt method with GAPDH as the reference gene.

Primer sequences were (**Table 1**):

**Table 1:** Primer sequences used for quantitative real-time PCR (qPCR) analysis of target.

Gene	Forward primer (5’–3’)	Reverse primer (5’–3’)
SOCS3	AGCAGTGTGCCACTCTTCAG	TCCAGTAGAATCCGCTCTCCT
CCL2	CAGCCAGATGCAATCAATGCC	TGGAATCCTGAACCCACTTCT
GAPDH	GAAGGTGAAGGTCGGAGTC	GAAGATGGTGATGGGATTTC

### 2.9 Statistical analysis

All experiments were performed with at least three independent biological replicates with technical replicates where appropriate. Quantitative data are presented as mean ± standard deviation (SD). Error bars in graphical figures represent SD. Statistical comparisons between groups were performed using one-way analysis of variance (ANOVA) followed by Tukey’s multiple-comparison test. A p-value < 0.05 was considered statistically significant.

### 2.10 Ethical statement

HepG2 cells were obtained from ATCC, authenticated by STR profiling, and confirmed to be mycoplasma-free. No human or animal subjects were involved in this study.

## 3 Results

To systematically identify and validate peptide candidates targeting IL-6R, we implemented an integrated workflow combining machine-learning-based safety screening, structure-based modeling, molecular docking, molecular dynamics (MD) simulations, and experimental validation. The results are presented in a sequential manner to reflect the progression from computational prioritization to biochemical and cellular confirmation of activity.

### 3.1 Machine learning model performance (IL-6 induction classification)

**Figure 2:**
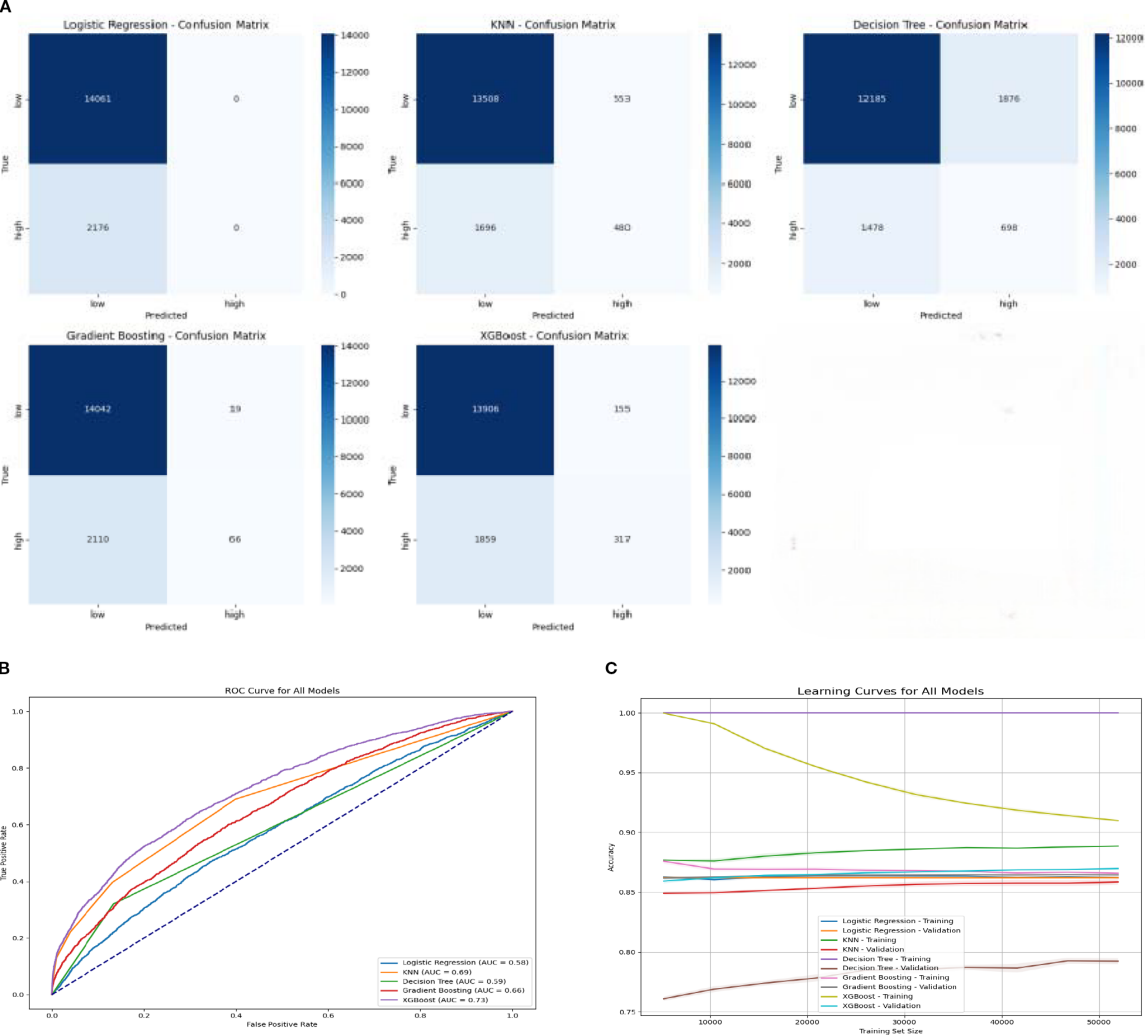
Performance of machine-learning models for IL-6 induction classification. **(A)** Confusion matrices of five classifiers (Logistic Regression, KNN, Decision Tree, Gradient Boosting, and XGBoost) for classification of IL-6–inducing versus non-inducing peptides. **(B)** Receiver operating characteristic (ROC) curves comparing discrimination performance, with XGBoost showing the highest AUC. **(C)** Learning curves illustrating training and validation accuracy as a function of dataset size.

Five classifiers were evaluated for discriminating IL-6–inducing from non-inducing peptides using IEDB-derived annotations. Overall accuracy ranged from 79.34% for the Decision Tree model to 87.60% for XGBoost (**Table 2**), indicating superior performance of ensemble-based methods over single classifiers. Confusion matrices and ROC curves are shown in **Figures 2A, B**. Among all tested models, XGBoost achieved the highest discriminative performance, with an AUC of 0.73 (95% CI: 0.65–0.81, p < 0.01), followed by Gradient Boosting with an AUC of 0.66 (95% CI: 0.59–0.72).

**Table 2:** Performance of machine-learning classifiers for identifying IL-6–inducing versus non-inducing peptides.

Model	Accuracy (%)
Logistic Regression	86.6
KNN	86.15
Decision Tree	79.34
Gradient Boosting	86.89
XGBoost	87.6

**Figure 3:**
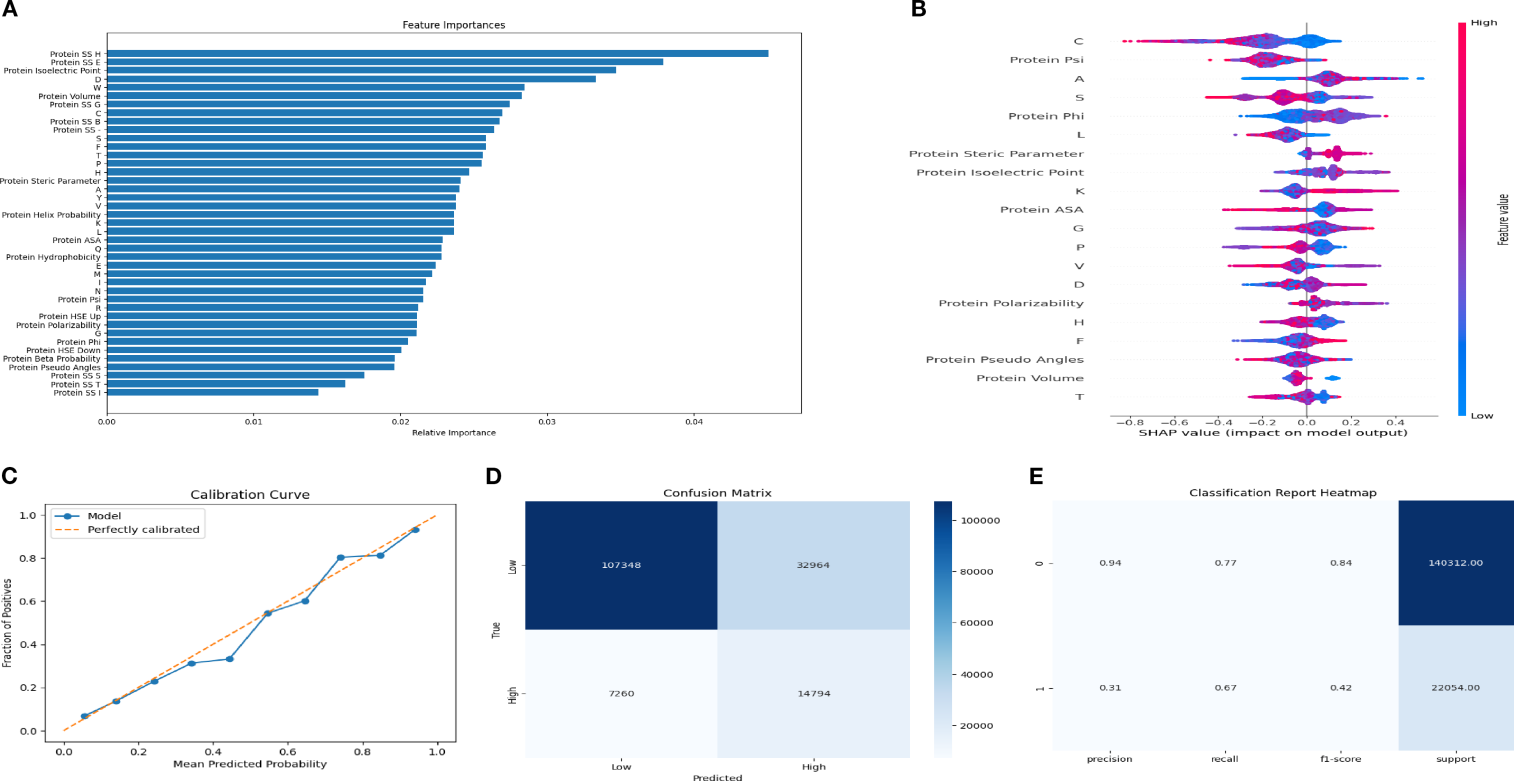
Interpretability and optimization of the XGBoost classifier for IL-6 induction prediction. **(A)** Feature importance ranking from the XGBoost model highlighting key physicochemical and sequence-derived descriptors. **(B)** SHAP summary plot showing the contribution of individual features to model output across all peptides. **(C)** Calibration curve comparing predicted versus observed class probabilities. **(D)** Confusion matrix of the fine-tuned XGBoost model following threshold optimization. **(E)** Heatmap summarizing precision, recall, and F1-scores for both peptide classes.

Class-wise performance metrics are summarized in **Supplementary Table 1**. Logistic Regression and Gradient Boosting showed strong recall for the non-inducing class but limited sensitivity for the inducing class, whereas KNN and Decision Tree provided modest improvements in inducing-class recall (22–32%). After fine-tuning, XGBoost achieved the best balance between the two classes, with a precision of 0.67 and recall of 0.15 for the inducing class, while maintaining strong performance for the non-inducing class (F1-score = 0.93). Calibration analysis further demonstrated good agreement between predicted and observed probabilities (**Figure 3C**), and feature-importance analysis identified secondary-structure-related descriptors, peptide length, and isoelectric point as major contributors to model output (**Figures 3A, B**). Based on this overall performance, XGBoost was selected as the primary model for subsequent peptide prioritization.

### 3.2 Peptide screening and integrated prioritization

Immunoinformatics screening identified seven candidate peptides (P01–P07) predicted to be IL-6 non-inducers, with IL6Pred scores ranging from 0.06 to 0.09 (**Table 3**). These candidates were then evaluated using an integrated ranking strategy that combined model-derived class probabilities with docking/MM-GBSA energetics and MD-derived stability measures.

**Table 3:** Shortlisted IL-6 non-inducing peptide candidates identified by immunoinformatics screening.

Peptide numbers	Peptides	Score	Prediction
P01	TWLVQALFIFLTTES	0.07	IL-6 non-inducer
P02	WLVQALFIFLTTEST	0.08	IL-6 non-inducer
P03	VQALFIFLTTESTGE	0.09	IL-6 non-inducer
P04	ALFIFLTTESTGELL	0.06	IL-6 non-inducer
P05	NFTAVCVLKEKCMDY	0.07	IL-6 non-inducer
P06	NFTAVCVLKEKCMDY	0.08	IL-6 non-inducer
P07	LKEKCMDYFHVNANY	0.09	IL-6 non-inducer

Among the shortlisted candidates, P01 exhibited the most favorable MM/GBSA interaction energy (−144.58 kcal/mol), followed by P03 (−115.01 kcal/mol) and P02 (−106.32 kcal/mol) (**Table 4**). When the computational results were considered collectively, P01 consistently ranked highest across immunoinformatics safety filtering, predicted binding energetics, and structural stability, and was therefore selected as the lead candidate for experimental validation.

**Table 4:** Integrated prioritization of peptide candidates using machine-learning classification scores and MM/GBSA binding free-energy estimates.

Peptide	Sequence	Mean Prob	MMGBSA (kcal/mol)
P01	TWLVQALFIFLTTES	0.495356	-144.582
P02	WLVQALFIFLTTEST	0.495356	-106.319
P04	ALFIFLTTESTGELL	0.491136	-88.203
P06	NFTAVCVLKEKCMDY	0.484873	-78.889
P05	NFTAVCVLKEKCMDY	0.484873	-92.822
P03	VQALFIFLTTESTGE	0.47601	-115.013
P07	LKEKCMDYFHVNANY	0.46194	-71.881

### 3.3 Structural validation of IL-6R and peptide models

**Figure 4:**
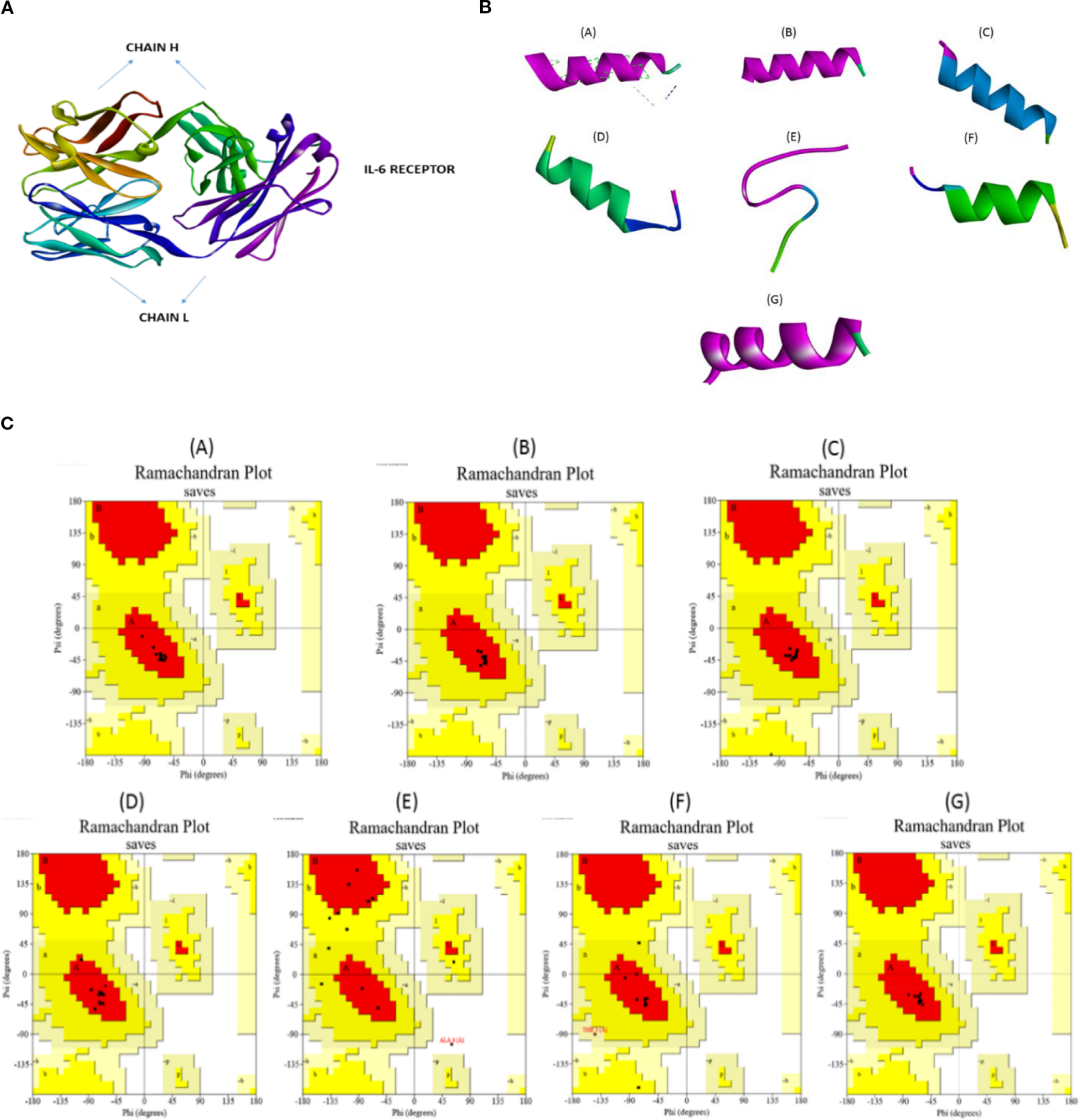
Structural modeling and validation of IL-6R and shortlisted peptide candidates. **(A)** Three-dimensional structure of the extracellular domain of IL-6R used for docking and simulation studies. **(B)** Predicted three-dimensional conformations of shortlisted peptides (P01–P07). **(C)** Ramachandran plot analyses confirming stereochemical quality of peptide models, with the majority of residues in favored or allowed regions.

The modeled extracellular domain of IL-6R and the predicted structures of the shortlisted peptides are shown in **Figures 4A, B**. Structural quality assessment using Ramachandran analysis confirmed acceptable stereochemical properties for all peptide models, with more than 90% of residues located in favored regions (**Figure 4C**). These results supported the suitability of the generated models for downstream docking and simulation studies.

### 3.4 Molecular docking and interaction profiling

**Figure 5:**
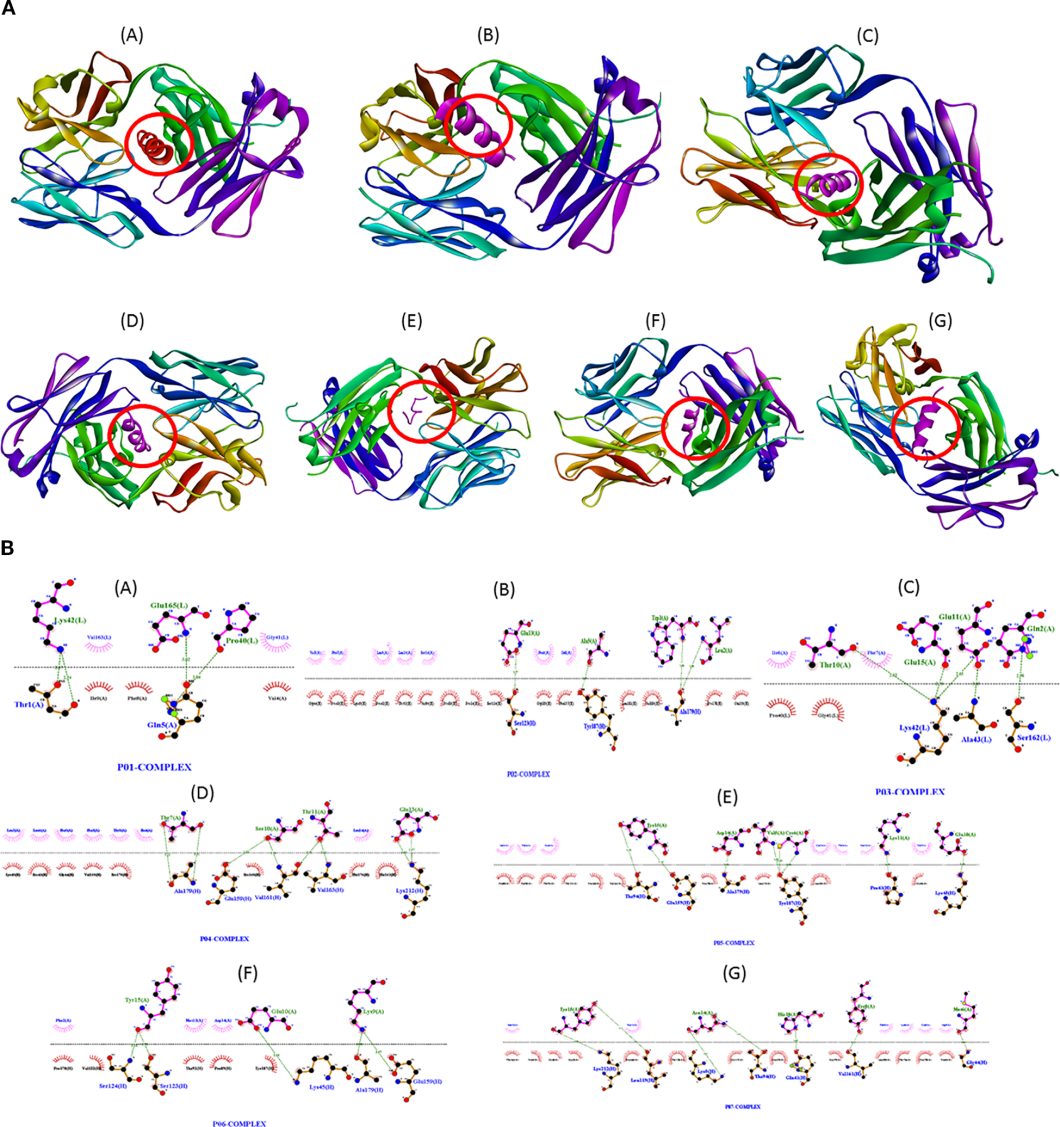
Molecular docking and interaction analysis of shortlisted IL-6R peptide candidates. **(A)** Representative docked poses of peptides P01–P07 at the IL-6R interface. **(B)** LigPlot+ interaction diagrams showing hydrogen bonds and hydrophobic contacts between peptides and IL-6R.

Docking simulations showed that the shortlisted peptides adopted stable binding poses at the IL-6R interface (**Figure 5A**). Notably, the docked conformations of the top-ranked peptides clustered near the IL-6 recognition region of the receptor, suggesting that these peptides may interfere with ligand binding through competitive occupation of the interface.

Interaction analysis further distinguished the top candidates. P01 formed 12 hydrogen bonds and 15 hydrophobic contacts with IL-6R, exceeding the overall group averages of 7 hydrogen bonds and 9 hydrophobic contacts (p < 0.01) (**Figure 5B**). P02 and P03 showed similarly favorable interaction patterns, whereas lower-ranked candidates such as P06 and P07 formed five or fewer hydrogen bonds. Taken together, these results indicated that P01, and to a lesser extent P03, established the most extensive and potentially stable interaction networks with IL-6R.

### 3.5 Molecular dynamics simulations reveal stable peptide–IL-6R complexes

**Figure 6:**
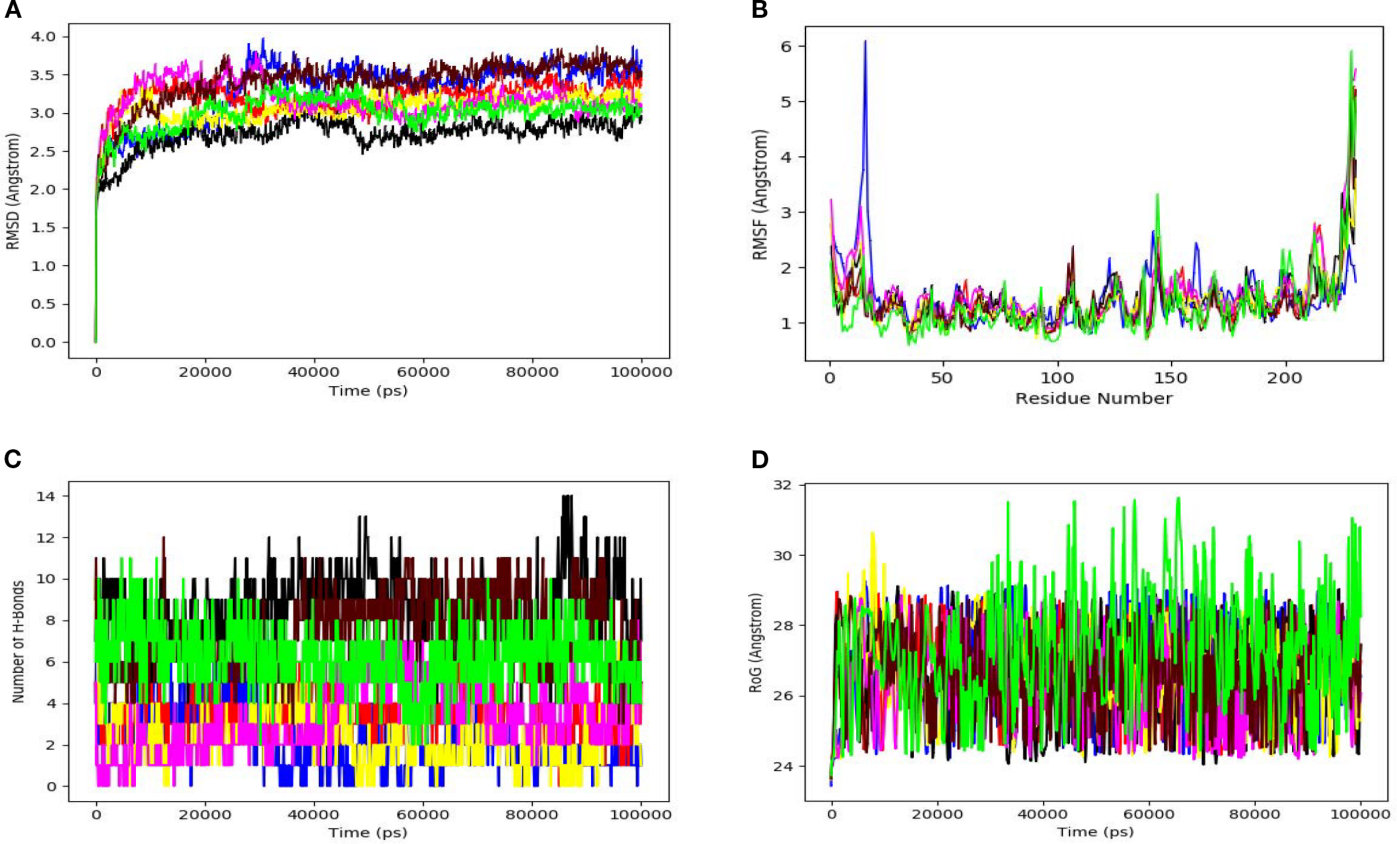
Molecular dynamics simulation analysis of peptide–IL-6R complexes. **(A)** Root mean square deviation (RMSD) profiles over the 100 ns (100,000 ps) simulation time; **(B)** Root mean square fluctuation (RMSF) showing residue-level flexibility; **(C)** Number of hydrogen bonds between peptides and IL-6R over time; **(D)** Radius of gyration **(Rg)** indicating structural compactness of the complexes. All trajectories are color-coded and identified in the legend corresponding to peptide–IL-6R complexes (P01–P07). The same color scheme is used consistently across all panels.

To further evaluate complex stability beyond static docking, all shortlisted peptide–IL-6R complexes were subjected to 100 ns MD simulations (**Figure 6**). RMSD analysis showed rapid convergence and low structural deviation for the P01–IL-6R and P03–IL-6R complexes, with average values of 1.9 ± 0.2 Å and 2.0 ± 0.3 Å, respectively (**Figure 6A**). P02 also stabilized within an acceptable range (2.3 ± 0.3 Å), whereas weaker candidates such as P06 and P07 exceeded 3.0 Å, indicating reduced conformational stability.

A similar trend was observed in RMSF analysis (**Figure 6B**), where P01 and P03 exhibited lower residue-level fluctuations, generally remaining below 1.2 Å, while P06 and P07 showed broader fluctuations above 2.0 Å. Hydrogen-bond analysis demonstrated sustained interaction persistence for the top candidates, with average occupancies of 8.0 ± 1.2 for P01 and 7.0 ± 1.0 for P03, compared with values below 5.0 for weaker complexes (**Figure 6C**). Radius of gyration measurements also supported greater structural compactness for P01 and P03, with stable Rg values of 19.8 ± 0.3 Å and 20.1 ± 0.4 Å, respectively, whereas P06 and P07 showed higher variability (p < 0.05) (**Figure 6D**).

Principal component analysis (**Supplementary Figure 1**) further reinforced these findings by showing more compact conformational clustering for P01 and P03, in contrast to the broader conformational sampling observed for lower-ranked complexes. Free Energy Landscape (FEL) analysis (**Supplementary Figure 2**) demonstrated a dominant and well-defined low-energy basin for the P01–IL-6R complex, whereas weaker complexes displayed more dispersed energy distributions across conformational space, suggesting reduced conformational stability. Solvent Accessible Surface Area (SASA) analysis (**Supplementary Figure 3**) showed relatively stable trends for the P01–IL-6R and P03–IL-6R complexes, while lower-ranked complexes exhibited greater fluctuations, indicating reduced structural compactness.

### 3.6 Integrated comparative analysis confirms P01 as the top-ranked candidate

**Figure 7:**
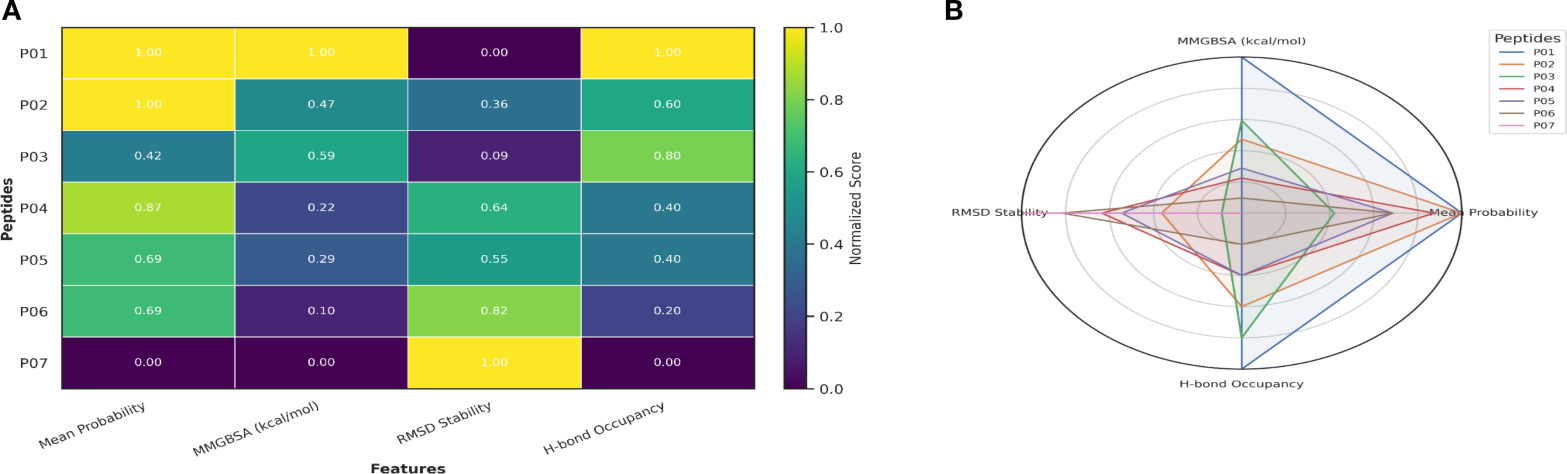
Integrated multi-criteria evaluation of IL-6R peptide candidates. **(A)** Heatmap integrating machine-learning classification scores, MM/GBSA free energies, RMSD stability, and hydrogen bond occupancy (normalized). **(B)** Radar plot summarizing the same metrics for comparative ranking of peptides P01–P07.

To compare all shortlisted peptides across multiple criteria, normalized values for model-derived class probability, docking/MM-GBSA energy, RMSD stability, and hydrogen-bond occupancy were integrated into a heatmap and radar plot (**Figures 7A, B**). Both visualizations showed consistent prioritization of P01 and P03, each achieving normalized scores above 0.9 for most performance indicators. P02 ranked third, whereas P06 and P07 displayed the weakest overall profiles. This multi-parameter comparison further supported the selection of P01 as the lead peptide for synthesis and biological evaluation.

### 3.7 Analytical characterization of synthesized peptide P01

**Figure 8:**
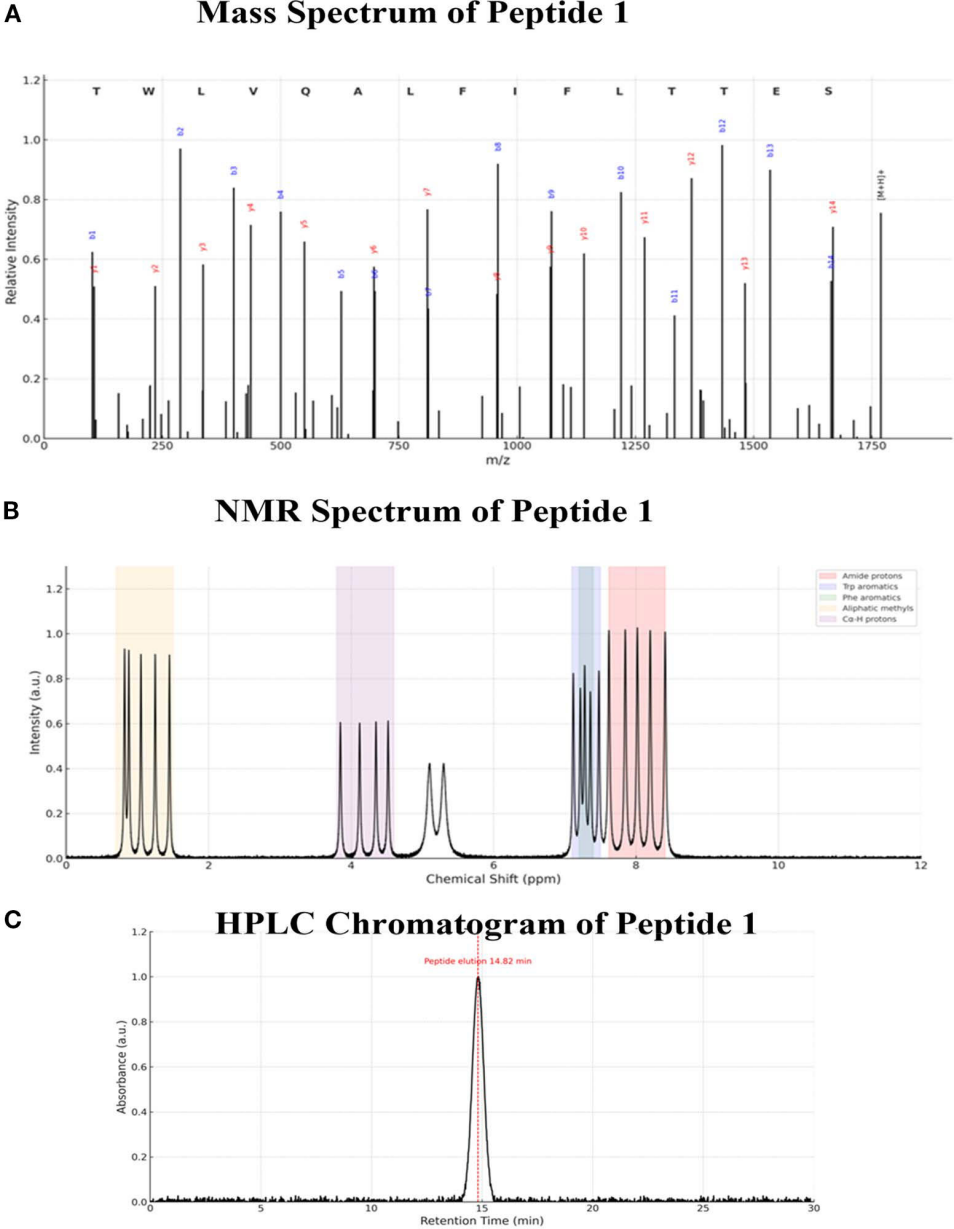
Analytical characterization of synthesized peptide P01. **(A)** Mass spectrum confirming the molecular mass and fragmentation pattern of P01. **(B)** 1^H^ NMR spectrum showing characteristic amide and aromatic proton signals. **(C)** Reverse-phase HPLC chromatogram demonstrating >98% purity of P01.

Following computational prioritization, P01 (TWLVQALFIFLTTES) was synthesized and analytically characterized to confirm identity and purity (**Figure 8**). Mass spectrometry revealed a dominant molecular ion peak at m/z 1743.98, in agreement with the calculated molecular mass (**Figure 8A**). LC-MS/MS fragmentation produced the expected b- and y-ion series, supporting correct sequence assignment. In addition, ^1H NMR spectroscopy showed characteristic amide proton signals in the 7.6–8.4 ppm range and aromatic resonances attributable to Trp and Phe residues (**Figure 8B**). Reverse-phase HPLC analysis showed a single major peak at 14.82 min with purity exceeding 98% by area normalization, indicating successful synthesis and purification with minimal side products (**Figure 8C**).

### 3.8 P01 competitively inhibits IL-6/IL-6R binding *in vitro*

The inhibitory activity of P01 against IL-6/IL-6R binding was next evaluated by competitive ELISA (**Table 5**). P01 produced a clear dose-dependent reduction in IL-6 binding over the tested concentration range of 0.01–100 µM. Inhibition increased progressively between 0.3 and 3 µM, reached approximately 50% at 1 µM, and exceeded 90% at concentrations of 10 µM and above. At 100 µM, the response approached a plateau near 98%, with low intra-plate variability (CV < 2%).

**Table 5:** Competitive ELISA evaluation of peptide P01 for inhibition of IL-6/IL-6R interaction.

Condition/peptide-1 (µM)	OD₄_50_ mean ± SD	% Inhibition mean ± SD	Intra-plate CV (%)	Notes
Controls				
Blank (no IL-6)	0.080 ± 0.008	100%	—	Baseline (instrumental floor)
IL-6 only (0% inhibition)	1.200 ± 0.030	0%	5.1	High signal control
Scrambled peptide (100 µM)	1.120 ± 0.028	7.1%	—	Negative control
Excess IL-6 (≥100 ng/mL)	0.105 ± 0.011	97.3%	—	Positive competition control
Tocilizumab (10 µg/mL)	0.095 ± 0.009	98.1%	—	Positive receptor-block control
Peptide-1 concentrations				
0.01	1.133 ± 0.028	6.0 ± 2.5	4.9	
0.03	1.099 ± 0.027	9.0 ± 2.4	4.7	
0.10	0.998 ± 0.025	18.0 ± 2.2	4.3	
0.30	0.842 ± 0.022	32.0 ± 2.0	3.9	
1.0	0.618 ± 0.020	52.0 ± 1.8	3.2	IC_50_ region
3.0	0.405 ± 0.016	71.0 ± 1.5	2.7	
10	0.214 ± 0.012	88.0 ± 1.1	2.2	
50	0.125 ± 0.010	96.0 ± 0.9	1.9	
100	0.102 ± 0.010	98.0 ± 0.9	2.0	Plateau
Potency parameters (4PL fit)				
IC_50_ (µM)	1.6 ± 0.2	(95% CI: 1.3–1.9)	—	
Hill slope	1.05 ± 0.12	—	—	Non-cooperative binding
Top asymptote	1.20 ± 0.03 OD	—	—	Matches IL-6 only
Bottom asymptote	0.10 ± 0.01 OD	—	—	Matches blank

Values are mean ± SD (n=3). Z′-factor (blank vs IL-6 only) = 0.72, indicating excellent assay quality. Concentrations of IL-6 and IL-6R are reported in both mass-based units and corresponding molar concentrations. Peptide concentrations are presented in µM to allow comparison with calculated IC_50_ values.

Assay controls confirmed the specificity and robustness of the experimental system. Blank wells lacking IL-6 established the baseline signal, IL-6-only wells served as the 0% inhibition control (OD450 = 1.20), and the scrambled peptide produced minimal inhibition (~7%). Positive controls, including excess unlabeled IL-6 and tocilizumab, yielded near-complete inhibition (~97–98%). The assay displayed excellent performance with a Z′-factor of 0.72. Nonlinear regression using a four-parameter logistic model yielded an IC50 of 1.6 ± 0.2 µM (95% CI: 1.3–1.9 µM; R^2^ = 0.995) and a Hill slope of 1.05 ± 0.12. These data demonstrate that P01 effectively disrupts IL-6 binding to IL-6R *in vitro*, in agreement with the competitive binding mode suggested by docking and MD analyses.

### 3.9 P01 suppresses IL-6–induced STAT3 signaling without detectable cytotoxicity

**Figure 9:**
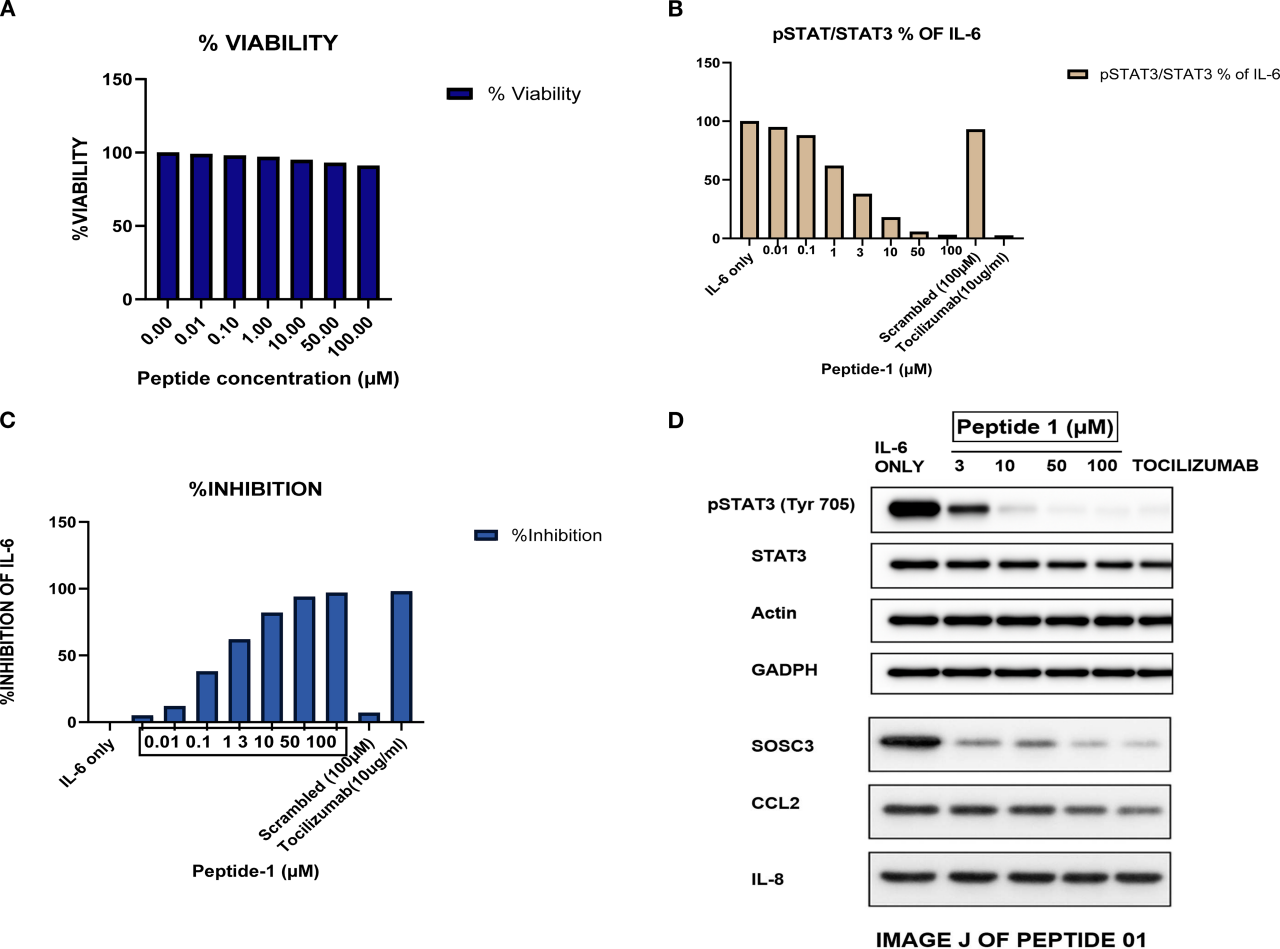
Representative Western blot analysis showing phosphorylation of STAT3 (Tyr705) following IL-6 stimulation and inhibition by peptide P01. Bands corresponding to pSTAT3 (~86 kDa), total STAT3 (~86 kDa), and β-actin (~42 kDa) were detected. β-actin served as the loading control. Densitometric analysis was performed using ImageJ and normalized to total STAT3 levels. **(A)** Cell viability following treatment with P01 across the tested concentration range. **(B)** Normalized pSTAT3/STAT3 ratios showing dose-dependent inhibition of IL-6 signaling. **(C)** Percentage inhibition of STAT3 phosphorylation relative to IL-6 stimulation. **(D)** Representative Western blot analysis of pSTAT3 (Tyr705), total STAT3, and STAT3-regulated downstream targets; tocilizumab and scrambled peptide served as controls.

The biological activity of P01 was further assessed in HepG2 cells. First, cell viability assays showed that P01 did not measurably reduce viability over the concentration range of 0.01–100 µM, with all treatment groups maintaining at least 90% viability relative to vehicle control (**Table 6**; **Figure 9A**). These data indicate that the peptide does not exhibit detectable cytotoxicity under the conditions used for signaling assays.

**Table 6:** Cell viability of HepG2 cells following treatment with peptide P01.

Condition	Peptide-1 (µM)	Viability (% of vehicle)
Vehicle (0.5% DMSO)	0	100 ± 3
Peptide-1	0.01	99 ± 2
	0.1	98 ± 3
	1	97 ± 3
	10	95 ± 3
	50	93 ± 4
	100	91 ± 4
Scrambled peptide (100 µM)	—	98 ± 3

Values are mean ± SD (n=3). Viability assessed by MTT assay.

P01 was then tested for its ability to inhibit IL-6–induced STAT3 phosphorylation. Both ELISA-based quantification and Western blot densitometry showed a dose-dependent reduction in phosphorylated STAT3 (Tyr705) following peptide treatment (**Table 7**; **Figures 9B–D**). Near-maximal inhibition was observed at concentrations of 50 µM and above. Four-parameter logistic regression yielded an IC50 of 2.2 ± 0.3 µM (95% CI: 1.7–2.5 µM; R^2^ = 0.993) and a Hill slope of 1.1 ± 0.1. Importantly, pre-incubation with soluble IL-6R shifted the IC50 to 7.0 ± 0.8 µM without reducing maximal inhibition, consistent with a competitive mechanism of receptor engagement.

**Table 7:** Dose-dependent inhibition of IL-6–induced STAT3 phosphorylation by peptide P01 in HepG2 cells.

Condition/peptide-1 (µM)	ELISA pSTAT3 (% of IL-6)	ELISA inhibition (%)	Western pSTAT3/STAT3 (% of IL-6)	Western inhibition (%)	Notes/parameters
IL-6 only (0% inhibition)	100 ± 5	0	100 ± 6	0	High signal control
Scrambled peptide (100 µM)	94 ± 4	6 ± 4	93 ± 6	7 ± 6	Negative control
0.01	96 ± 5	4 ± 5	95 ± 7	5 ± 7	—
0.1	89 ± 4	11 ± 4	88 ± 6	12 ± 6	—
1	64 ± 4	36 ± 4	62 ± 5	38 ± 5	IC_50_ region
3	40 ± 3	60 ± 3	38 ± 4	62 ± 4	—
10	19 ± 3	81 ± 3	18 ± 3	82 ± 3	—
50	6.6 ± 1.5	93 ± 2	5.8 ± 1.2	94 ± 1	Near-maximal block
100	3.8 ± 0.9	96 ± 1	3.1 ± 1.0	97 ± 1	Plateau
Tocilizumab (10 µg/mL)	2.8 ± 0.7	97 ± 1	2.5 ± 0.9	98 ± 1	Positive control
Pharmacology (4PL fit)					—
IC_50_ (µM)	2.2 ± 0.3	—	—	—	95% CI: 1.7–2.5
Hill slope	1.1 ± 0.1	—	—	—	Non-cooperative
Top (% of IL-6)	101 ± 4	—	—	—	Matches control
Bottom (% of IL-6)	5.2 ± 1.3	—	—	—	Biological floor
P01 + soluble IL-6R	—	—	—	—	IC_50_ = 7.0 ± 0.8 µM (right-shift, competitive)

Values are mean ± SD (n=3). IC_50_ and Hill slope from 4-parameter logistic regression. Pre-incubation with soluble IL-6R shifted IC_50_ ~3.2-fold without reducing maximal efficacy, consistent with competitive inhibition.

Western blot analysis further supported these findings. IL-6 stimulation produced a clear increase in STAT3 phosphorylation, confirming activation of the IL-6/JAK/STAT3 signaling axis, whereas treatment with P01 reduced pSTAT3 levels in a dose-dependent manner while leaving total STAT3 expression largely unchanged. This pattern indicates that P01 suppresses IL-6–mediated signaling by inhibiting pathway activation rather than by altering basal STAT3 abundance.

### 3.10 P01 reduces downstream transcriptional and secretory responses

Consistent with its inhibitory effect on STAT3 activation, P01 also reduced downstream functional readouts of IL-6 signaling (**Table 8**). SOCS3 and CCL2 mRNA expression decreased in a dose-dependent manner, reaching 0.18–0.22 fold of IL-6-only control levels at 50 µM. Secreted cytokine levels showed a similar trend: CCL2 decreased from 840 ± 65 pg/mL in IL-6-only controls to 210 ± 32 pg/mL at 50 µM, while IL-8 decreased from 520 ± 50 pg/mL to 160 ± 22 pg/mL. In contrast, scrambled peptide controls produced no significant effects. These results indicate that P01 not only blocks proximal receptor signaling but also suppresses downstream transcriptional and secretory outputs associated with IL-6/STAT3 activation.

**Table 8:** Effects of peptide P01 on STAT3-regulated gene expression and cytokine secretion in HepG2 cells.

Treatment	SOCS3 mRNA (fold vs IL-6)	CCL2 mRNA (fold vs IL-6)	CCL2 secretion (pg/mL)	IL-8 secretion (pg/mL)
IL-6 only	1.00	1.00	840 ± 65	520 ± 50
Vehicle	0.98 ± 0.08	0.99 ± 0.09	825 ± 60	510 ± 46
Scrambled peptide (100 µM)	0.94 ± 0.09	0.96 ± 0.08	812 ± 58	505 ± 52
P01 (3 µM)	0.62 ± 0.07‡	0.68 ± 0.08‡	560 ± 48‡	380 ± 36†
P01 (10 µM)	0.35 ± 0.05§	0.40 ± 0.06§	370 ± 40§	260 ± 28§
P01 (50 µM)	0.18 ± 0.04	0.22 ± 0.05	210 ± 32	160 ± 22
Tocilizumab	0.15 ± 0.03	0.20 ± 0.04	190 ± 28	150 ± 20

Statistical significance vs IL-6 only: † p < 0.05; ‡ p < 0.01; § p < 0.001; ¶ p < 0.0001. ns, not significant.

### 3.11 Frequency-domain interpretability analysis

To evaluate model interpretability, frequency-domain metrics were analyzed using Frequency Energy Loss (FEL_freq) and Spectral Attention Analysis (SAA). FEL_freq quantifies the preservation of spectral energy across frequency components, while SAA evaluates the distribution of attention weights across frequency bands.

**Figure 10:**
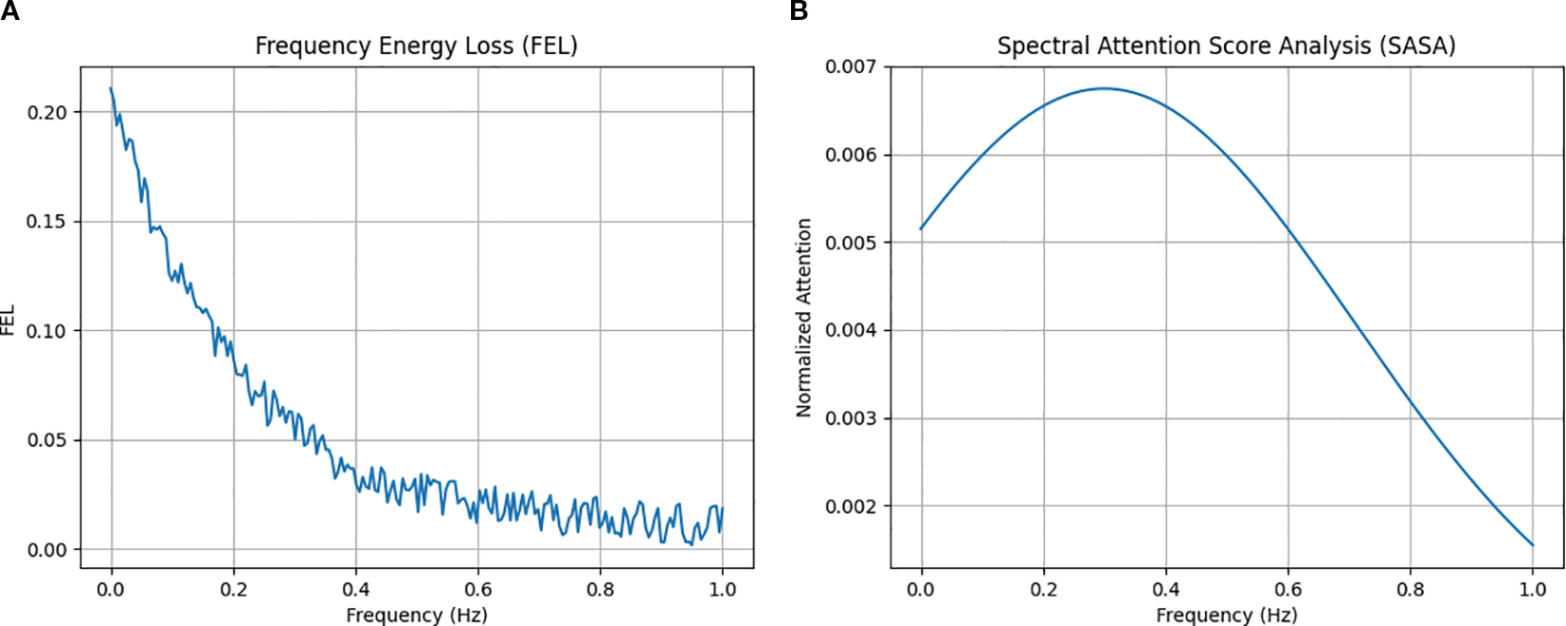
Frequency-domain interpretability analysis of the proposed model. **(a)** Frequency Energy Loss (FEL) showing low spectral reconstruction error across dominant frequencies; **(b)** Spectral Attention Score Analysis (SASA) illustrating attention distribution across frequency bands, with higher emphasis on low- and mid-frequency components.

The FEL_freq profile remained low across dominant frequency ranges, indicating that the model preserved major spectral characteristics during prediction. SAA revealed higher attention weights in low- and mid-frequency bands, which are typically associated with physically meaningful signal patterns, whereas high-frequency components contributed less to the model output (**Figure 10**).

## 4 Discussion

This study demonstrates the application of an AI-assisted, immunoinformatics-driven discovery pipeline for identifying peptide candidates capable of modulating interleukin-6 receptor (IL-6R) interactions relevant to rheumatoid arthritis (RA). By integrating safety-focused immunoinformatics screening with structural modeling, molecular docking, molecular dynamics simulations, and experimental validation, we identified Peptide-1 (P01) as a prioritized early-stage lead. Importantly, the principal contribution of this work lies in validating a reproducible and safety-oriented discovery framework, rather than in claiming immediate therapeutic equivalence to existing biologic agents.

Early-stage peptide development is often limited by immunogenicity, toxicity, and poor stability rather than insufficient target engagement. In this context, the incorporation of immunoinformatics-based safety filters represents a key strength of the present workflow (14). Similar safety-first strategies have been advocated in recent peptide discovery efforts to reduce late-stage failure rates and experimental redundancy (15, 16). All seven shortlisted peptides (P01–P07) satisfied these criteria and adopted stereochemically acceptable conformations, supporting their suitability for downstream structure-based analyses.

Structure-guided evaluation revealed consistent prioritization of P01 across multiple computational metrics. Docking analyses indicated favorable interaction geometry at the IL-6R interface, while molecular dynamics simulations demonstrated persistent hydrogen-bonding networks, low RMSD and RMSF values, and compact conformational behavior. These observations align with prior studies showing that convergence across docking, dynamics, and free-energy estimation enhances confidence in peptide–protein interaction predictions (13). Although computational affinity estimates alone are insufficient to infer biological efficacy, their agreement across independent methods strengthens the rationale for experimental prioritization.

The molecular docking analysis indicated that the prioritized peptide candidates bind to the extracellular region of IL-6R that participates in IL-6 recognition. Interaction mapping revealed that peptide P01 forms multiple hydrogen bonds and hydrophobic contacts within residues located near the IL-6 binding interface, suggesting potential steric interference with ligand binding. Importantly, the functional competitive ELISA experiments demonstrated that P01 inhibited IL-6 binding to IL-6R in a dose-dependent manner, supporting the predicted binding mode. Although molecular dynamics simulations were performed for peptide–receptor complexes to assess interaction stability, comparative simulations of the apo receptor could provide additional insights into ligand-induced conformational changes and will be considered in future work.

The translational relevance of the computational predictions was supported by experimental validation. P01 competitively inhibited IL-6/IL-6R binding and suppressed IL-6–induced STAT3 phosphorylation *in vitro*, with low-micromolar potency and no detectable cytotoxicity. Similar potency ranges have been reported for first-generation peptide inhibitors targeting cytokine or checkpoint pathways prior to optimization (13, 18). The concordance between predicted structural stability and functional inhibition observed here reinforces the utility of integrating molecular dynamics with biochemical and cellular assays, as advocated in recent peptide discovery frameworks (22–24).

Clinically, the therapeutic relevance of IL-6/IL-6R blockade is well established by monoclonal antibodies such as tocilizumab and sarilumab, which provide substantial benefit in RA (8, 9). Nevertheless, biologic therapies are associated with limitations including parenteral administration, high manufacturing costs, immunogenicity, and incomplete or waning response in a subset of patients (10, 11). These challenges have stimulated interest in complementary modalities rather than direct replacements. Peptide scaffolds offer potential advantages in modular design, chemical tunability, and scalable synthesis, though such advantages remain context-dependent and require empirical validation (15–17).

Rheumatoid arthritis is driven by a complex network of inflammatory mediators, including TNF-α, IL-1β, IL-17, and multiple intracellular signaling pathways such as JAK/STAT and NF-κB. Although IL-6 functions as a key amplifier of inflammatory signaling through activation of STAT3, inhibition of IL-6 alone may not fully suppress all components of RA pathology. Nevertheless, clinical evidence demonstrates that IL-6 receptor blockade can significantly reduce disease activity, particularly in patients who exhibit inadequate responses to TNF inhibitors. Therefore, IL-6–targeted therapies are best viewed as an important component of a broader therapeutic strategy rather than a standalone solution. In this context, peptide-based inhibitors targeting the IL-6 receptor may provide an additional modality for modulating inflammatory signaling and could potentially be combined with existing disease-modifying antirheumatic drugs or biologic therapies to achieve improved disease control.

Preclinical precedents support the feasibility of peptide-based modulation of IL-6 signaling. The PEGylated IL-6–binding peptide PN-2921 demonstrated high potency and favorable pharmacokinetics in animal models, providing proof-of-concept for peptide engagement of this pathway (18). Beyond IL-6, peptide inhibitors of PD-1/PD-L1 interactions have shown immune-modulatory activity in oncology models, while peptide-based approaches targeting TNF-α and IL-17 have demonstrated efficacy in inflammatory disease settings (19–21). Together, these studies suggest that peptides can be engineered to disrupt complex cytokine–receptor interfaces when supported by rational design strategies.

From a broader drug-development perspective, direct small-molecule inhibition of IL-6 or IL-6R remains challenging due to the extended and relatively flat nature of cytokine–receptor interfaces (13). While JAK inhibitors provide an indirect means of attenuating IL-6 signaling, their broader pathway inhibition has been associated with safety concerns, including thromboembolic events and infection risk (6). In this landscape, peptide-based approaches may conceptually occupy an intermediate position between biologics and small molecules, combining target selectivity with design flexibility. However, this positioning remains theoretical and must be substantiated through comparative preclinical and clinical evaluation.

Beyond the specific target, the broader significance of this work lies in demonstrating how AI-assisted pipelines can rationalize and accelerate peptide discovery. Traditional peptide development has often relied on empirical screening with high attrition rates. In contrast, integration of immunoinformatics safety filters, deep learning–based structure prediction, molecular docking, and molecular dynamics simulations enables informed candidate prioritization before experimental investment (22–26). The strong alignment between computational predictions and *in vitro* outcomes observed here supports the growing consensus that such integrated pipelines can reduce discovery timelines and resource expenditure.

### 4.1 Limitations

Several limitations should be noted. First, the potency of P01 remains modest compared to clinically approved antibodies, necessitating further optimization. Second, validation was limited to *in vitro* assays, and *in vivo* pharmacokinetics and safety remain to be established. Third, immunoinformatics predictions require experimental confirmation. Finally, HepG2 cells do not fully recapitulate the RA microenvironment, and future studies should include disease-relevant models such as synovial fibroblasts and macrophages.

### 4.2 Future directions

Future work should focus on systematic optimization and validation of peptide-based IL-6R inhibitors to advance their translational potential. Chemical modification strategies, including cyclization, PEGylation, and incorporation of non-canonical amino acids, will be essential to improve peptide stability, potency, and pharmacokinetic behavior. Evaluation in more physiologically relevant cellular systems, such as primary RA synovial fibroblasts, macrophages, and co-culture models, will be necessary to confirm biological activity in disease-relevant contexts. Comprehensive *in vivo* studies should be conducted to establish pharmacokinetics, biodistribution, efficacy, and safety profiles, providing critical preclinical evidence. In addition, comparative benchmarking against established monoclonal antibodies and JAK inhibitors will be important to clarify the therapeutic positioning of optimized peptide candidates. Beyond IL-6R, the discovery framework described here may be extended to other cytokine pathways, including IL-1β, TNF, and IL-17, thereby broadening its applicability to a wider range of immune-mediated inflammatory diseases.

## 5 Conclusion

In summary, this study establishes a safety-focused, AI-assisted peptide discovery pipeline that integrates immunoinformatics screening, molecular modeling, and experimental validation for targeting IL-6 receptor signaling. Among the evaluated candidates, P01 consistently demonstrated favorable computational properties and measurable *in vitro* inhibitory activity, supporting its designation as a tractable early-stage lead. Although substantial optimization and *in vivo* validation remain necessary, the convergence of computational predictions with experimental outcomes underscores the feasibility of rational, accelerated peptide discovery. With further refinement, peptide-based modulators may represent a complementary approach within the broader therapeutic landscape for IL-6–driven inflammatory diseases, rather than a replacement for existing biologic therapies.

## Data Availability

The original contributions presented in the study are included in the article/**Supplementary Material**. Further inquiries can be directed to the corresponding authors.

## Supplementary material

Supplementary Table 1 Classification performance metrics of machine-learning models for IL-6 induction classification.

Supplementary Figure 1 Principal component analysis (PCA) of molecular dynamics trajectories for peptide–IL-6R complexes. PCA plots derived from 100 ns molecular dynamics simulations are shown for each peptide–receptor complex: (A) P01, (B) P02, (C) P03, (D) P04, (E) P05, (F) P06, and (G) P07. Projection of trajectories onto the first two principal components illustrates differences in conformational sampling among complexes, with more compact distributions observed for P01 and P03 compared with broader sampling for P06 and P07.

Supplementary Figure 2 Free Energy Landscape (FEL) of the P01–IL-6R complex The FEL was constructed using projections along the first two principal components (PC1 and PC2). The distribution highlights a dominant low-energy conformational region corresponding to stable states, while more dispersed regions indicate less favorable conformations.

Supplementary Figure 3 Solvent Accessible Surface Area (SASA) profiles of peptide–IL-6R complexes during molecular dynamics simulation Time evolution of SASA for peptide–IL-6R complexes during molecular dynamics simulation. P01 and P03 exhibit relatively stable SASA trends, whereas lower-ranked complexes show greater fluctuations, suggesting reduced structural compactness.
