# Supplementary material for: From in silico to in vitro: discovery and validation of peptide modulators of IL-6/IL-6R signaling in rheumatoid arthritis
Source: Front Immunol. 2026 Apr 29;17:1794095. doi: 10.3389/fimmu.2026.1794095 (PMC13168006; doi:10.3389/fimmu.2026.1794095)
Supplement: Supplementary Table 1 — Classification performance metrics of machine-learning models for IL-6 induction classification. [file SupplementaryFile1.docx]

**Supplementary**

**Supplementary Table S1.** **Classification performance metrics of machine-learning models for IL-6 induction classification.**

| **Model** | **Precision (low)** | **Recall (low)** | **F1-Score (low)** | **Precision (high)** | **Recall (high)** | **F1-Score (high)** |
| --- | --- | --- | --- | --- | --- | --- |
| Logistic Regression | 0.87 | 1.00 | 0.93 | 0.00 | 0.00 | 0.00 |
| KNN | 0.89 | 0.96 | 0.92 | 0.46 | 0.22 | 0.30 |
| Decision Tree | 0.89 | 0.87 | 0.88 | 0.27 | 0.32 | 0.29 |
| Gradient Boosting | 0.87 | 1.00 | 0.93 | 0.78 | 0.03 | 0.06 |
| XGBoost | 0.88 | 0.99 | 0.93 | 0.67 | 0.15 | 0.24 |

**
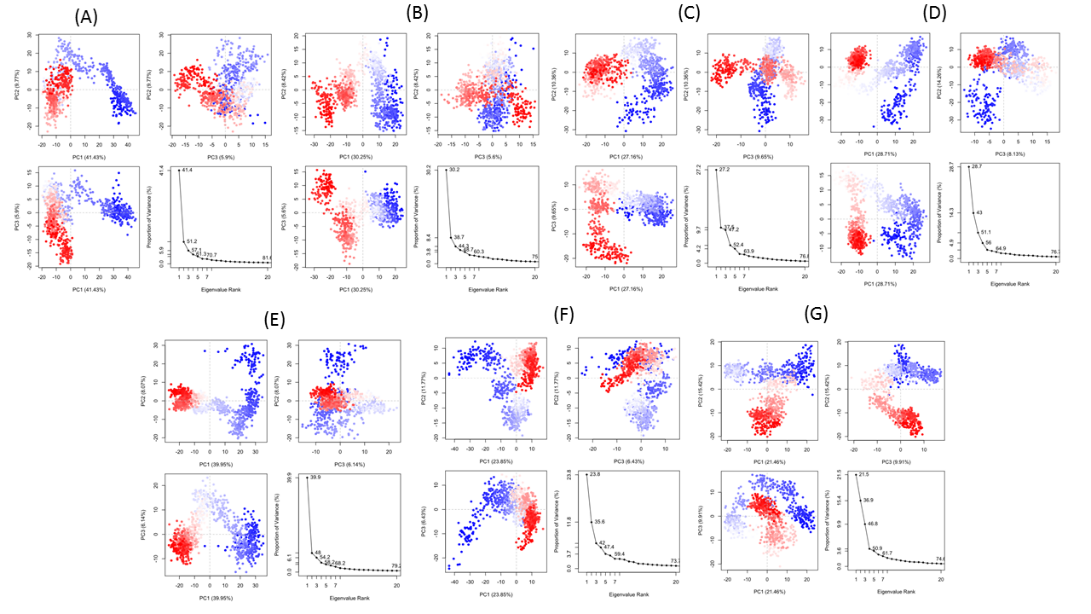
**

**Supplementary Figure S1. Principal component analysis (PCA) of molecular dynamics trajectories for peptide–IL-6R complexes.**

PCA plots derived from 100 ns molecular dynamics simulations are shown for each peptide–receptor complex: (A) P01, (B) P02, (C) P03, (D) P04, (E) P05, (F) P06, and (G) P07. Projection of trajectories onto the first two principal components illustrates differences in conformational sampling among complexes, with more compact distributions observed for P01 and P03 compared with broader sampling for P06 and P07.


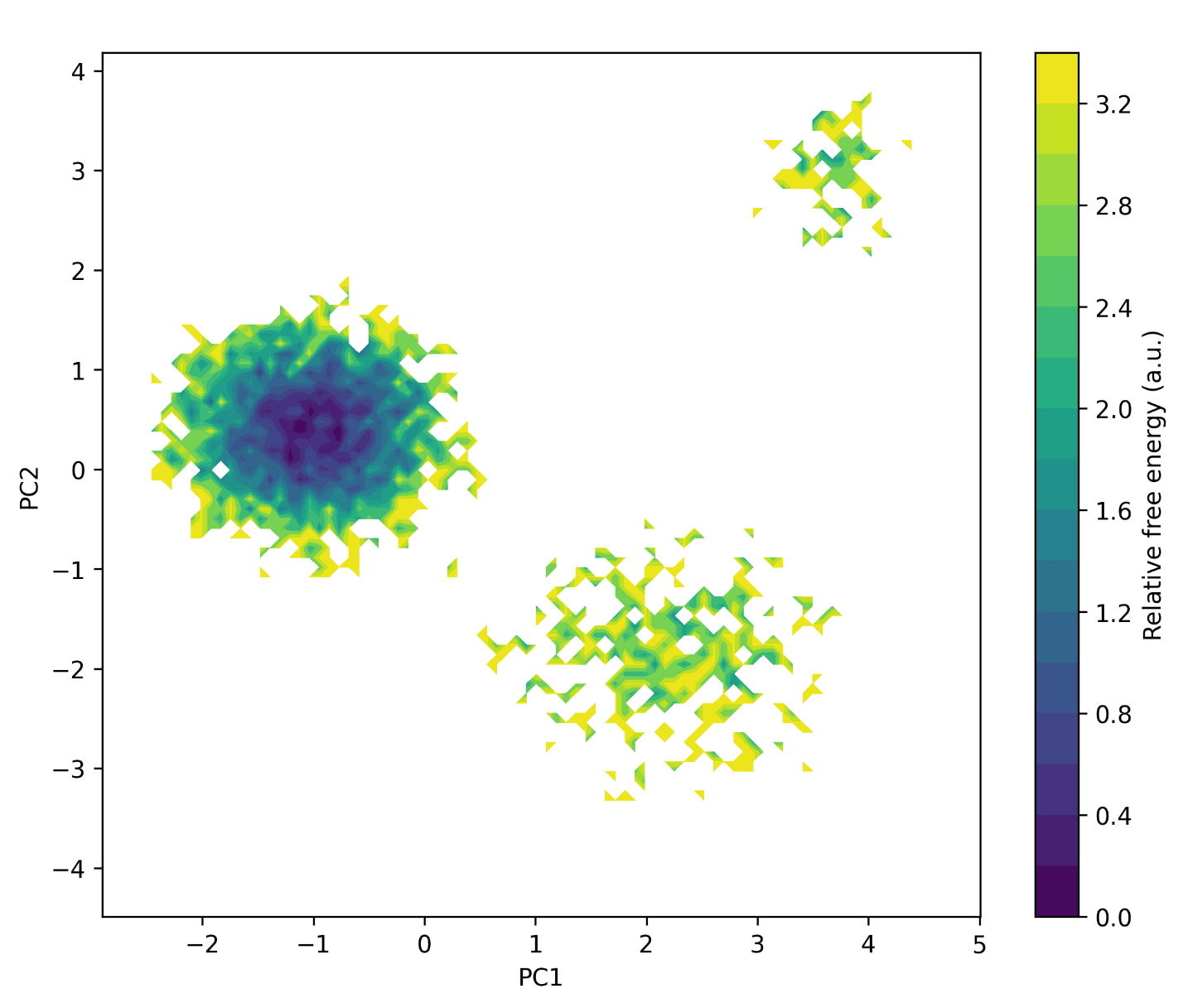


**Supplementary Figure S2. Free Energy Landscape (FEL) of the P01–IL-6R complex**

The FEL was constructed using projections along the first two principal components (PC1 and PC2). The distribution highlights a dominant low-energy conformational region corresponding to stable states, while more dispersed regions indicate less favorable conformations.


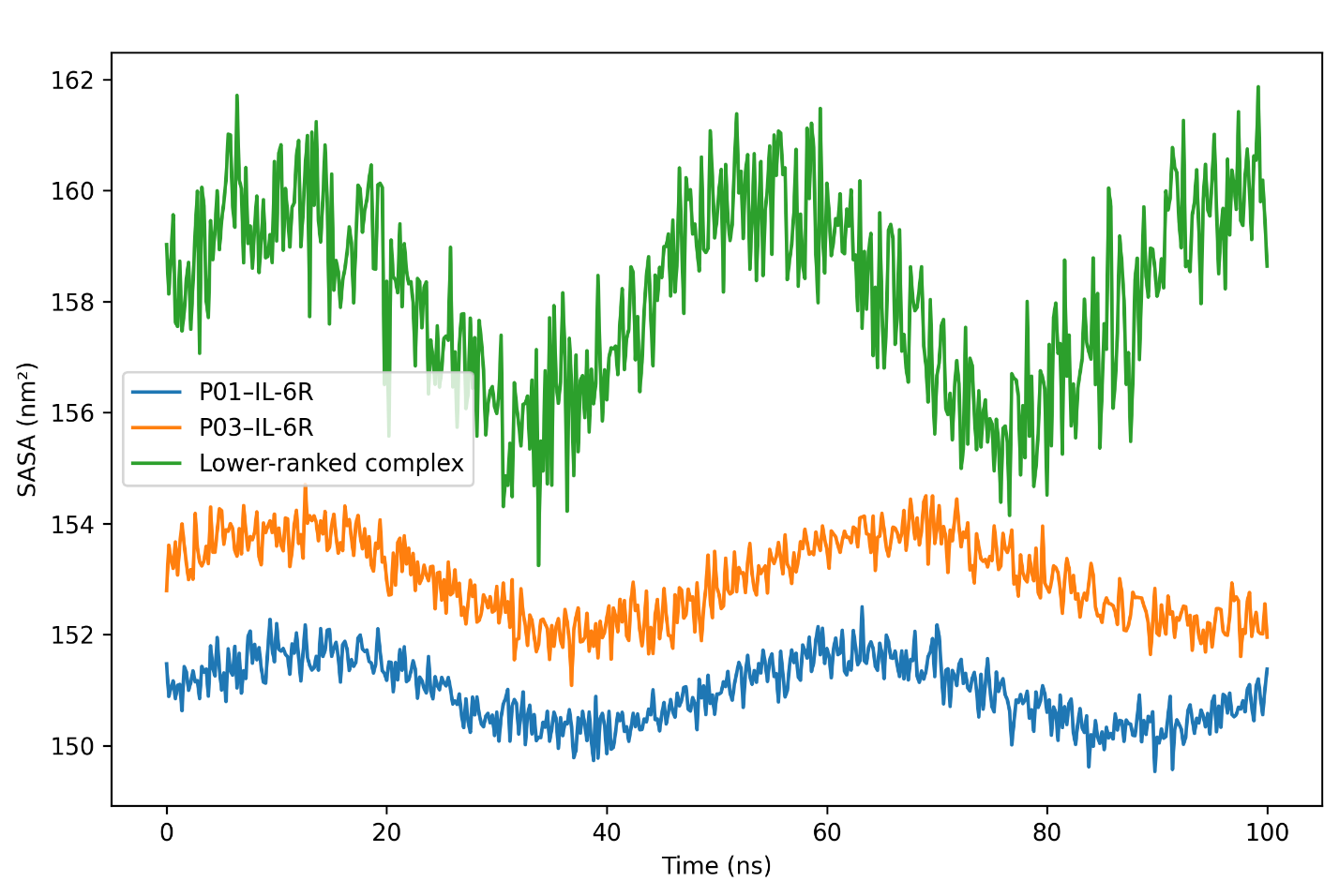


**Supplementary Figure S3. Solvent Accessible Surface Area (SASA) profiles of peptide–IL-6R complexes during molecular dynamics simulation**

Time evolution of SASA for peptide–IL-6R complexes during molecular dynamics simulation. P01 and P03 exhibit relatively stable SASA trends, whereas lower-ranked complexes show greater fluctuations, suggesting reduced structural compactness.
